# JAZ8 Interacts With VirE3 Attenuating Agrobacterium Mediated Root Tumorigenesis

**DOI:** 10.3389/fpls.2021.685533

**Published:** 2021-11-19

**Authors:** Shijuan Li, Bingliang Xu, Xiaolei Niu, Xiang Lu, Jianping Cheng, Meiliang Zhou, Paul J. J. Hooykaas

**Affiliations:** ^1^College of Plant Protection, Gansu Agricultural University, Lanzhou, China; ^2^Hainan Key Laboratory for Sustainable Utilization of Tropical Bioresource, College of Tropical Crops, Hainan University, Haikou, China; ^3^College of Agriculture, Guizhou University, Guiyang, China; ^4^Institute of Crop Sciences, Chinese Academy of Agricultural Sciences, Beijing, China; ^5^Department of Molecular and Developmental Genetics, Institute of Biology, Leiden University, Leiden, Netherlands

**Keywords:** *Agrobacterium tumefaciens*, *Arabidopsis thaliana*, VirE3, JAZ8, jasmonates

## Abstract

*Agrobacterium tumefaciens* can cause crown gall tumors by transferring both an oncogenic piece of DNA (T-DNA) and several effector proteins into a wide range of host plants. For the translocated effector VirE3 multiple functions have been reported. It acts as a transcription factor in the nucleus binding to the *Arabidopsis thaliana* pBrp TFIIB-like protein to activate the expression of *VBF*, an F-box protein involved in degradation of the VirE2 and VIP1 proteins, facilitating *Agrobacterium*-mediated transformation. Also VirE3 has been found at the plasma membrane, where it could interact with VirE2. Here, we identified AtJAZ8 in a yeast two-hybrid screening with VirE3 as a bait and confirmed the interaction by pull-down and bimolecular fluorescence complementation assays. We also found that the deletion of *virE3* reduced *Agrobacterium* virulence in a root tumor assay. Overexpression of *virE3* in Arabidopsis enhanced tumorigenesis, whereas overexpression of *AtJAZ8* in Arabidopsis significantly decreased the numbers of tumors formed. Further experiments demonstrated that AtJAZ8 inhibited the activity of VirE3 as a plant transcriptional regulator, and overexpression of *AtJAZ8* in Arabidopsis activated *AtPR1* gene expression while it repressed the expression of *AtPDF1.2*. Conversely, overexpression of *virE3* in Arabidopsis suppressed the expression of *AtPR1* whereas activated the expression of *AtPDF1.2*. Our results proposed a novel mechanism of counter defense signaling pathways used by *Agrobacterium*, suggesting that VirE3 and JAZ8 may antagonistically modulate the salicylic acid/jasmonic acid (SA/JA)-mediated plant defense signaling response during *Agrobacterium* infection.

## Introduction

The plant pathogen *Agrobacterium tumefaciens*, a soil-borne gram negative bacterium, provokes crown gall disease in dicotyledonous plant species, which is characterized by neoplastic growth at infection sites (McCullen and Binns, 2006; Guo et al., 2019). During infection, *Agrobacterium* transfers a single-stranded DNA segment from its tumor-inducing (Ti) plasmid, the T-DNA, into the plant cell, where it integrates into the chromosomes (Beijersbergen and Hooykaas, 1993; Gelvin, 2000, 2003, 2021; Citovsky et al., 2007; Guo et al., 2019). Expression of the oncogenic genes located on T-DNA in transformed plant cells promotes uncontrolled cell proliferation and results in the formation of crown gall tumors (Nester et al., 1984). Under laboratory conditions, *Agrobacterium* is also able to transform non-plant organisms, such as the yeast *Saccharomyces cerevisiae* and fungi (Bundock and Hooykaas, 1996; Piers et al., 1996; de Groot et al., 1998). By taking advantage of this unique ability of interkingdom gene transfer, *Agrobacterium* has been widely used in creating transgenic plants and fungi for research and biotechnology through a method known as the *Agrobacterium*-mediated transformation (AMT).

Besides the T-region, the virulence (vir) region in the Ti plasmid embraces a series of genes that are essential for AMT, which facilitate T-DNA processing, delivery, and integration into host cell nucleus. Within *Agrobacterium*, the virulence protein VirD2, in conjunction with VirD1, recognizes and generates nicks in the border sequence surrounding the T-DNA region, releasing the T-strand from the Ti plasmid (Yanofsky et al., 1986; Herrera-Estrella et al., 1988). VirD2 remains covalently attached to the 5′ end of the T-strand in *Agrobacterium*, and then the VirD2–T-strand complex is delivered into host cells through a type IV secretion system (T4SS) composed of 11 VirB proteins and VirD4 (Young and Nester, 1988; Christie et al., 2005; Li and Christie, 2018). In host cells, the nuclear localization signal (NLS) of the VirD2 protein pilots the T-strand to the nucleus (Howard et al., 1992; Shurvinton et al., 1992; Tinland et al., 1992; Rossi et al., 1993). In addition, some of the virulence proteins, nowadays referred to as effector proteins, including VirE2, VirE3, VirD5, and VirF are directly translocated into host cells *via* the T4SS independent of the T-strand (Vergunst, 2000; Schrammeijer, 2003). VirE2 is a single-strand DNA binding protein which is supposed to coat the T-strand in the host cytoplasm to form a T-complex, protecting the T-strand from degradation by host nucleases (Citovsky et al., 1989; Gelvin, 1998; Abu-Arish et al., 2004). VirE2 can interact with VIP1, a host bZIP transcription factor, which relocalizes from the cytoplasm to the nucleus to activate defense-related genes upon *Agrobacterium* infection (Tzfira, 2001; Djamei et al., 2007). It has been reported that VIP1 assists in the nuclear entry of the T-complex and facilitates transformation (Tzfira, 2001; Li et al., 2005; Djamei et al., 2007). However, the importance of VIP in AMT is controversial, as no difference in transformation susceptibility between wild-type and *vip1-1* mutant was observed, and a recent report pointed out that VIP1 and its homologs are not required for AMT (Shi et al., 2014; Lapham et al., 2018). The VirF protein is a host–range factor (Melchers et al., 1990; Jarchow et al., 1991) and contains an F-box motif by which it interacts with plant Arabidopsis Skp1–like (ASK1) to form an Skp1–Cullin–F-box (SCF) E3 ubiquitin ligase in the host cell (Jarchow et al., 1991; Schrammeijer et al., 2001). The SCF^*virF*^ is thought to use the host ubiquitin-26S proteasome system to promote proteolysis of VirE2, VIP1, and some other host proteins in nucleus (Tzfira et al., 2004; Zaltsman et al., 2012; García-Cano et al., 2018). Degradation of VirE2 and VIP1 uncoats the T-complex and thus, may enable integration of T-DNA into the host genome (Tzfira et al., 2004). In addition, proteasomal degradation of Arabidopsis transcription factor VIP1 and VFP4 may dampen the defense response of the host cells (Djamei et al., 2007; García-Cano et al., 2018). Some plant species like Arabidopsis, encode an endogenous F-box protein “VBF,” which can functionally replace VirF, and therefore, do not require VirF for transformation (Zaltsman et al., 2010).

VirE3 is another translocated effector that has been shown to interact with VirE2 and bind to plant importin-a, possibly acting as an “adapter” molecule between VirE2 and importin-a to assist the nuclear import of the T-complex, thereby mimicking the function of VIP1 (Lacroix et al., 2004). More recently, an interaction between VirE3 and VirE2 was found to take place at the plasma membrane, suggesting that VirE3 may be involved in the early stage of T-complex formation (Li et al., 2018). Besides, VirE3 can act as a transcriptional activator in the nucleus to modulate the plant gene expression profile through interaction with pBrp, a plant specific general transcription factor belonging to the TFIIB family (García-Rodríguez et al., 2006). One of the genes strongly induced by VirE3 was pointed out to be the gene encoding VBF, thus explaining why simultaneous deletion of *virF* and *virE3* results in a much stronger deficiency of tumorigenicity than observed in the single mutant (Niu et al., 2015). Another gene highly induced by VirE3 was *NIMIN1*, which operates as a negative regulator by binding to NPR1 and reduces salicylic acid (SA)-mediated *PR1* gene expression and attenuates the defense response (Weigel et al., 2005; Niu et al., 2015). Here, we found that *Agrobacterium* VirE3 can interact with Arabidopsis JAZ8 and that overexpression of *virE3* promotes tumor formation, whereas *JAZ8* overexpression makes roots recalcitrant to tumorigenesis by *Agrobacterium*. JAZ proteins are best known as repressors of the jasmonate response, which recruit general corepressors to prevent the transcriptional activity of MYC transcription factors (Pauwels and Goossens, 2011; Howe et al., 2018). We found that the activity of VirE3 as a plant transcription regulator was repressed by JAZ8. The jasmonic acid (JA)-regulated *PDF1.2* gene expression was elevated in the *virE3*-overexpressor plant line, but the SA-mediated *PR1* gene expression was reduced in the *virE3*-overexpressor plant line. Our results suggest the VirE3 and JAZ8 may antagonistically modulate each other’s activity, and that *Agrobacterium* uses VirE3 to shift the plant defense response during AMT from SA signaling to JA signaling, which is less deleterious for biotrophic pathogens.

## Materials and Methods

### Plasmid Constructs

Supplementary Tables 1–3 list the primer sequences, plasmids, and strains used in this study, respectively. The *JAZ8* coding sequence was amplified by PCR using cDNA of Col-0 *Arabidopsis thaliana* with the primer set JAZ8-1302-F and JAZ8-1302-R. The PCR fragment was inserted into pCAMBIA1302 by homologous recombination, yielding *JAZ8* overexpression construct JAZ8-1302. The *JAZ8* sequence was amplified by PCR on plasmid JAZ8-1302 using primers listed in Supplementary Table 1 for JAZ8-pAS2.1, JAZ8-ZIM-pAS2.1, JAZ8NT-pAS2.1, JAZ8CT-pAS2.1, JAZ8-pMAL-c5X, JAZ8-cYFP, and pRT101-JAZ8 constructs. The PCR fragments were digested and inserted into pAS2.1, pMAL-c5X, pRTL2-HAYC, and pRT101, yielding corresponding constructs.

The *virE3* coding sequence was amplified by PCR on plasmid pRT101-VirE3 (Niu et al., 2015) using primers listed in Supplementary Table 1 for virE3-pACT, virE3-pGEX6P-1, and virE3-nYFP constructs. The resulting PCR fragment was digested and inserted into the pACT2, pGEX-6P-1, and pRTL2-EEYN vector, yielding corresponding constructs. All constructs were verified by sequencing.

### Plant Materials, Growth Conditions, and Plant Transformation

The genetic background of ecotype Col-0 was used as wild type *Arabidopsis thaliana*. JAZ8-1302 (pCAMBIA1302) construct and 1,302 empty vector were used for Arabidopsis transformation. All plasmids used in this work are listed in Supplementary Table 2. The construct JAZ8-1302 and the empty vector 1,302 (used as control) were introduced into GV3101 by electroporation. Arabidopsis were transformed using the floral dip method (Clough and Bent, 1998) and grown at 21°C in a growth chamber (16-h light/8-h dark, 2,500 lux). Seeds from the dipped plants were harvested and planted on MS medium containing 30 mg L^–1^ hygromycin. The transgenic plants were verified by PCR of *Hph* gene. The positive seedlings were transferred to soil and grown until new seeds could be harvested. The TAM-*virE3* transgenic plant lines used in this study were from Niu et al. (2015).

### Yeast Two-Hybrid Assays

The truncated VirE3 (pASE3ΔC) without autoactivation activity was used as a bait in yeast two-hybrid screening (García-Rodríguez et al., 2006). The Arabidopsis cDNA library representing 5.7 × 10^5^ primary transformants was generated by Zhou (Zhou et al., 2017). The bait plasmid pASE3ΔC and Arabidopsis cDNA library was cotransformed into yeast strain PJ69-4A using the PEG-lithium acetate method (Gietz et al., 1992; James et al., 1996). Full-length JAZ8 (AT1G30135), JAZ8NT (aa 1–101), JAZ8CT (aa 101–127), and JAZ8-ZIM (aa 13–101) were cloned in pAS2.1 (acc. No. U30497). The corresponding vectors were cotransformed with empty pACT2 (acc. No. U29899) and pACT2-VirE3, respectively, to yeast strain PJ69-4A. Empty pAS2.1 and pACT2 were used as negative control. Yeast two-hybrid assays were conducted by the cotransformation of bait and prey plasmids into yeast strain PJ69-4A as described by Zhou (Zhou et al., 2017). Transformants were allowed to grow for 4 days at 28°C on synthetic defined (SD)-glucose plates lacking leucine, tryptophan, and histidine (-LWH). Then cells were incubated overnight in liquid SD–LWH medium and 4 μl of 100-fold dilutions were spotted on SD-LWH plate to grow for 4 days at 28°C. The experiments were repeated three times.

### *In vitro* Pull-Down Assay

The *JAZ8* coding sequence was cloned into pMAL-c5X through *Nde*I and *Bam*HI sites for fusion with MBP tag. Full-length CDS of *virE3* was cloned into pGEX6P-1 through *Bam*HI and *Sal*I sites for fusion with GST tag. Constructs JAZ8-pMAL-c5X and virE3-PGEX6P were transformed into *E. coli* strain BL21 (DE3) pLysS, separately and screened by 20 μg/ml chloramphenicol and 100 μg/ml carbenicillin. Protein expression of MBP-JAZ8 and GST-VirE3 were induced by 1 mM IPTG. The MBP-fusion proteins and GST-fusion proteins were extracted and immobilized onto MBP and GST agarose beads. For pull-down assays, proteins attached to the beads were subsequently examined by SDS-PAGE and detected with anti-MBP and anti-GST antibody, respectively. Detection was performed by incubating the blots in 8 ml western lighting reagent and then exposure to X-ray films.

### Bimolecular Fluorescence Complementation Assays

*virE3* was fused with the N-terminal part of the improved yellow fluorescent protein (YFP) in pRTL2-EEYN to produce VirE3-nYFP. *JAZ8* was fused with the C-terminal part of YFP in pRTL2-HAYC to produce JAZ8-cYFP (Bracha-Drori et al., 2004). VirE3-nYFP was transiently cotransformed with JAZ8-cYFP into the Arabidopsis leaf protoplast. Images of transfected protoplasts were acquired with a Leica DM IRBE confocal laser scanning microscope equipped an excitation at 514 nm and a band mission was detected at 530–600 nm. Microscopic images were analyzed using Image J software.

### *Agrobacterium-*Mediated Arabidopsis Root Tumor Assay

The *Agrobacterium* tumorigenic strain LBA 1010 (WT) and LBA 2564 (-*virE3*) were used for tumorigenesis assays on Arabidopsis Col-0 wild-type, *JAZ8*-OE, and *virE3*-OE plant lines. Arabidopsis root tumor assay was carried out as described previously with minor modifications (Valvekens et al., 1988; Czako et al., 1993). In brief, Arabidopsis seeds of Col-0, *JAZ8*-OE, and *virE3*-OE plant lines, respectively, were sterilized and incubated in liquid B5 medium at 4°C for 2 days. Then, the seeds in liquid B5 medium were incubated on a shaker (120 rpm) in growth room (21°C, 16 h light/8hrs dark, 2,000 lux) for 10 days. The root segments pooled from 10-day-old plant of Col-0, *JAZ8*-OE, and *virE3*-OE plants, respectively, were incubated with *Agrobacterium* LBA1010 and LBA2564 (OD_620_ = 0.2) for 2 min. The infected roots were cut into 4 mm (named explants) and dried on sterile filter paper, transferred to B5 agar plants containing 100 μM acetosyringone for cocultivation for 2 days in the growth room (25°C, 2,000 lux). After cocultivation, root explants were washed with liquid B5 and then transferred to B5 plates supplemented with 100 μg/ml timentin for 3 weeks until calli/tumors were formed. The percentage of roots that form tumors was calculated by counting tumors on 100 to 120 explants. Three independent replicates of 100 to 120 explants were used for each line.

### Arabidopsis Leaf Protoplast Transient Expression Assay

Plasmid *VBF*-promoter-GUS was obtained by inserting the promoter sequence of *VBF* (857bp) into plasmid GUSXX (Niu et al., 2015). Full length of *virE3* and *JAZ8* were inserted into plasmid pRT101. Plasmids carrying *VBF*-promoter-GUS and plasmids expressing VirE3 and/or JAZ8 were cotransformed into Arabidopsis Col-0 leaf protoplasts by polyethylene-glycol-mediated transformation as previously described (Schirawski et al., 2000). Cotransformation of pRT101 with GUSXX plasmids was used as control (Töpfer et al., 1987). Protoplasts were incubated at 25°C for 16 h and harvested by centrifugation. The GUS activity assays were performed as described (Niu et al., 2015). Relative expression levels of *virE3, AtJAZ8, AtPR1*, and *AtPDF1.2* were measured.

### Illumina mRNA-Seq Library Preparation and Sequencing

Arabidopsis seeds harvested from three independent *JAZ8*-OE and 1,302 plant lines were grown vertically on MS plates. RNA isolated from 10-day-old seedlings were used for Illumina sequencing. Illumina mRNA-seq libraries were generated and sequenced by Majorbio Cloud Platform^1^. The resulting libraries were checked and quantified. The libraries were multiplexed, clustered, and sequenced on an Illumina HiSeq 2000. Sequence reads of low quality and reads containing adaptor sequences were removed. To validate the RNA-seq results, total RNA was extracted from three independent *JAZ8*-OE and 1,302 plant lines using the same protocol for RNA-seq. cDNA was generated from total RNA. qRT-PCR was performed by Universal SYBR Green Master mix according to the manufacturer’s instructions. The relative amount of gene expression level was calculated using 2^–ΔΔ*Ct*^ method. *EF1a* (At5G09810) was used as the reference gene.

### Quantification of *AtPR1* and *AtPDF1.2* in *Agrobacterium* Infected Seedlings

Arabidopsis seeds of Col-0, *JAZ8-*OE, and *VirE3-*OE were sterilized and incubated in liquid B5 medium at 4°C for 2 days. Then, the seeds in liquid B5 medium were incubated on a shaker (120 rpm) in growth room (21°C, 16-h light/8-h dark, 2,000 lux) for 10 days. *Agrobacterium* strains LBA1100 and LBA2564 were preadjusted to OD600 = 0.2. Seedlings of the Col-0, *JAZ8-*OE, and *virE3-*OE were infected by *Agrobacterium* strains LBA1100 and LBA2564 for 2 min. The infected seedlings were taken out and immediately frozen in liquid nitrogen at different time points. RNA was extracted for qRT-PCR analysis. Quantitative RT-PCR analysis was carried out using Bio-Rad iCycler iQ5. The first strand of cDNA was synthesized by HiScript^®^ III RT SuperMix for qPCR (+ gDNA wiper) kit. Specific primers were designed for *AtPR1*, *AtPDF1.2*, and *EF1a* reference gene by primer 5. The primers are listed in Supplementary Table 1. The qRT-PCR reactions were as follows: 95°C 30 s; 95°C 10 s, 60°C 30 s, followed by 40 cycles. Relative expression levels of *AtPR1* and *AtPDF1.2* were calculated by 2^–ΔΔ*CT*^ methods. All dates are collected from three biological replicates.

### Statistical Analysis

The data were analyzed by Excel and SPSS software, and Duncan’s new complex range method was used to compare the differences among the treatments. *P* value < 0.05 was considered to be significant.

## Results

### VirE3 Interacts With JAZ8

**FIGURE 1:**
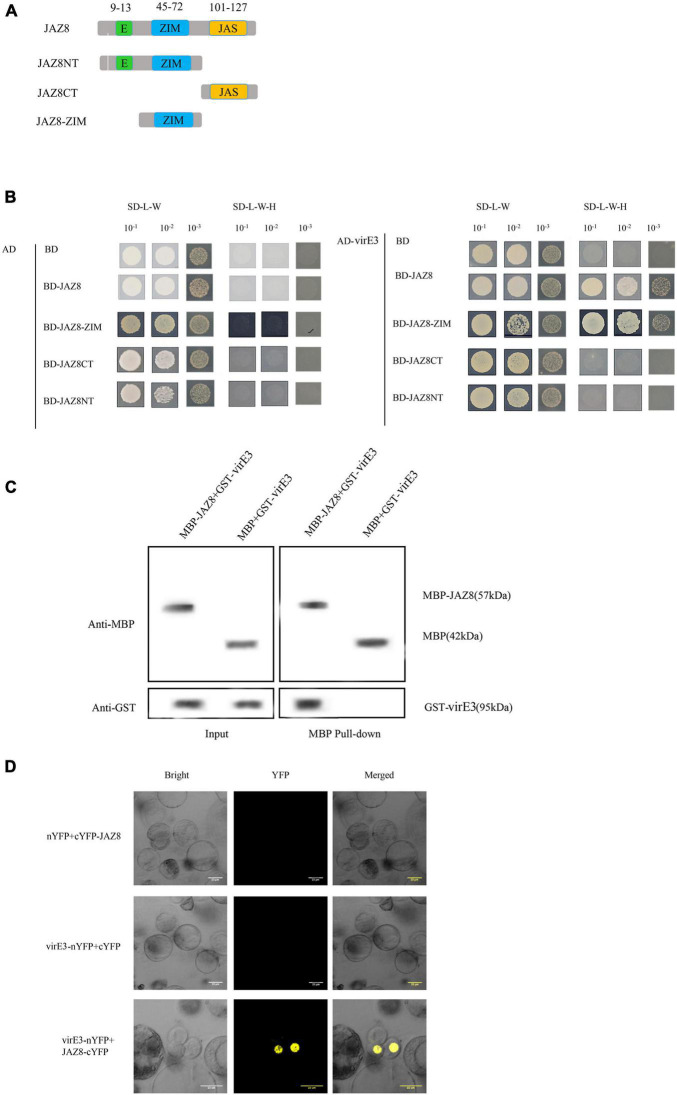
VirE3 interacts with AtJAZ8 *in vivo* and *in vitro.* **(A)** Schematic representation of JAZ8 constructs used for yeast two-hybrid assay. Numbers represent amino acid positions in full-length JAZ8. **(B)** Yeast two-hybrid assay. Full length of JAZ8 and the truncated fragments of JAZ8NT (aa 1–101), JAZ8CT (aa 101–127), JAZ8-ZIM (aa 13–101) were cloned into pAS2.1 (BD). The *virE3* was cloned into pACT2 (AD). The indicated prey and bait constructs were cotransformed into yeast strain PJ69-4A. The transformants were first screened on synthetic defined (SD) medium minus tryptophan and leucine (SD/-Trp-Leu) and the positive yeast cells were subcultured on SD medium minus tryptophan, leucine and histidine at 30°C for 4 days. pAS2.1 and pACT2 were used as the negative control. **(C)** Maltose-binding protein (MBP) pulldown assay. The MBP pull-down proteins were detected by Western blotting (WB) using anti-GST or anti-MBP antibody. MBP alone was used as the negative control. Input, protein samples without pull-down were analyzed by Western blotting. **(D)** Bimolecular fluorescence complementation (BIFC) assay. The constructs 35S::VirE3-nYFP and 35S::AtJAZ8-cYFP were transiently co-transformed into Arabidopsis protoplast and the transformed cells were observed under a confocal microscope. 35S::nYFP and 35S:: cYFP were used as negative control.

Early publications reported that VirE3 was bound with pBrp to facilitate AMT through modulating gene expression profiles in the host cell (García-Rodríguez et al., 2006). To gain further insight into the function of VirE3 within the host cells during *Agrobacterium* infection, we used the yeast two-hybrid system (Y2H) to screen for interactors of VirE3. Due to the transcriptional activation activity of the full-length VirE3 in yeast strain PJ69-4A, the truncated VirE3 (pASE3ΔC) without autoactivation activity (García-Rodríguez et al., 2006) was used as a bait and an Arabidopsis cDNA library (Zhou et al., 2017) was used as a prey. Five colonies were found to grow on minimal medium without histidine. Recovered prey plasmids were retransformed with pASE3ΔC into yeast strain PJ69-4A, and only two of these plasmids could grow on selective medium. From these candidate VirE3 interactors, only one cDNA sequence was in frame with the GAL4 activation domain and encoded the JAZ8 protein (AT1G30135). Our Y2H screening thus identified the Jasmonate ZIM-domain protein JAZ8 as a positive interactor of VirE3. To investigate which domain of JAZ8 was responsible for interaction with VirE3, we divided JAZ8 into N-terminal (JAZ8NT), ZIM domain containing (JAZ8-ZIM), and C-terminal fragments (JAZ8CT). JAZ8NT contained an EAR and a ZIM domain, whereas the JAZ8-ZIM and JAZ8CT only contained the ZIM domain and Jas domain, respectively (Figure 1A). We then cloned the full-length VirE3 into the activation domain (AD) and the different truncated versions of JAZ8 into the binding domain (BD) to verify the interaction in the Y2H system. We found that JAZ8, JAZ8NT, and JAZ8-ZIM interacted with VirE3 but not JAZ8CT. These results indicate that the ZIM domain of JAZ8 is responsible for the interaction between VirE3 and JAZ8 (Figure 1B). We further applied pull-down assays to verify the interaction between VirE3 and JAZ8 *in vitro*. GST-tagged VirE3 and maltose binding protein (MBP)-fused JAZ8 (MBP-JAZ8) were heterologously expressed in *E. coli* BL21 (DE3) cells and MBP-pull-down assays were performed using two fusion proteins. MBP-JAZ8 resin was incubated with GST-VirE3 and then separated on SDS-PAGE for immunoblotting with anti-GST antibody. As shown in Figure 1C, the negative control (MBP resin) was unable to pull-down GST-VirE3, whereas the GST-VirE3 was efficiently precipitated by MBP-JAZ8, indicating that VirE3 can physically interact with JAZ8 *in vitro*. We next used bimolecular fluorescence complementation (BiFC) assays to verify the interaction of VirE3 and JAZ8 proteins *in planta*. VirE3 was fused with the N-terminal part of the improved YFP in pRTL2-EEYN to produce VirE3-nYFP, and JAZ8 was fused with the C-terminal part of YFP in pRTL2-EEYN to produce JAZ8-cYFP. VirE3-nYFP was transiently coexpressed with JAZ8-cYFP in Arabidopsis protoplasts. We found that co-expression of VirE3-nYFP with JAZ8-cYFP resulted in strong YFP fluorescence in the nucleus of Arabidopsis mesophyll protoplasts (Figure 1D), whereas no YFP fluorescence was detected in negative controls (VirE3-nYFP coexpressed with cYFP or nYFP coexpressed with cYFP-JAZ8). Taken together, the Y2H assays, pull-down assays, and BiFC assays consistently demonstrate that VirE3 interacts with JAZ8 protein *in vitro* and *in vivo*.

### Effects of VirE3 and JAZ8 on *Agrobacterium*-Mediated Arabidopsis Root Tumorigenesis

**FIGURE 2:**
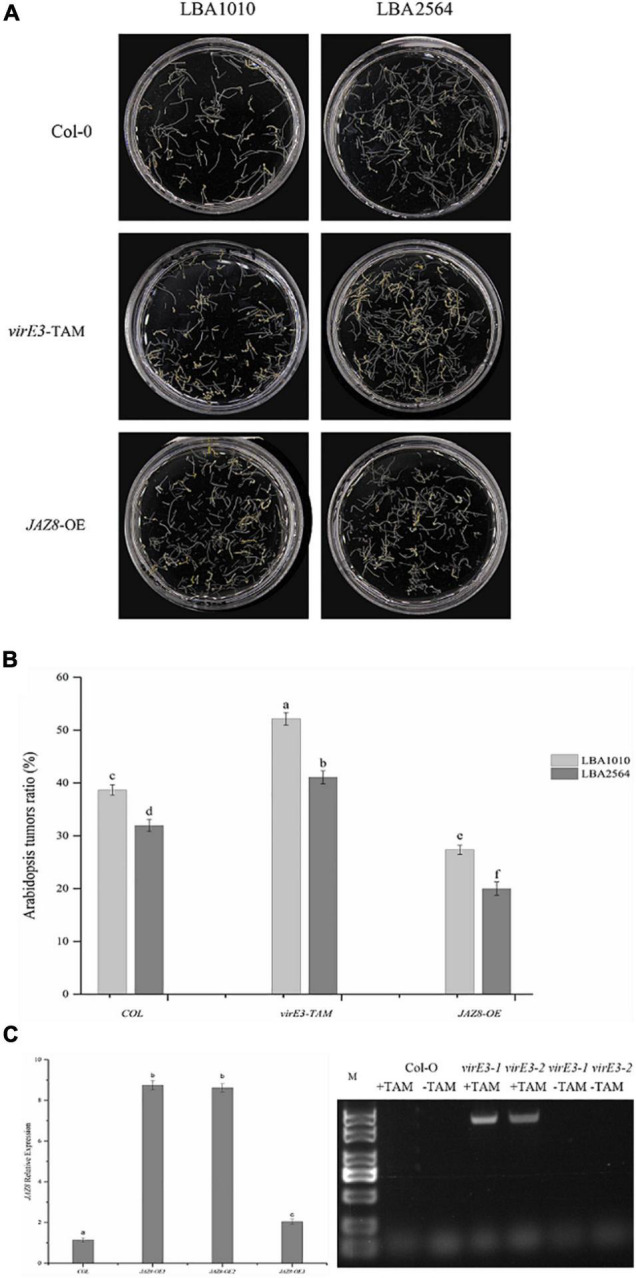
Efficiency of *Agrobacterium*-mediated root transformation in *virE3* and *JAZ8* overexpressor lines (*virE3*-TAM and *JAZ8*-OE lines) as compared with wild-type plants (Col-0) by *Agrobacterium* strain LBA1010 and *virE3* deletion mutant LBA2564. **(A)** Tumors developed on root explants. Root segments of Columbia, *virE3*-TAM1 and *JAZ8*-OE1 lines were infected with tumorigenic *Agrobacterium* strain LBA1010 and its *virE3* deletion mutant LBA2564. The root segments were photographed and scored 30 days after infection. **(B)** Quantification of the tumorigenicity showed in panel **(A)**. Error bars in B indicate SEM. Differences in tumorigenicity indicated by different letters are statistically significant (*P* values < 0.05) and by the same letter are not statistically significant. **(C)** Gene expression analysis of *JAZ8* and *virE3* in transgenic over-expressor plant lines.

It has been reported that VirE3 plays a role in tumorigenesis; however, mutation of *virE3* led to hardly any attenuation in tumor formation on the stems of six plant species including *Nicotiana glauca, Nicotiana tabacum, Helianthus annuus, Lycopersicon esculentum, Kalanchoe tubiflora*, and *Kalanchoe daigramontiana* (García-Rodríguez et al., 2006). To better understand the role of VirE3 in tumorigenesis, the *Agrobacterium* wild-type strain (LBA1010) and a *virE3* deletion strain (LBA2564) were compared in more quantitative root transformation assays on Arabidopsis Col-0. As shown in Figure 2A, the *virE3* mutant developed a lower number of tumors compared with LBA1010 (average 32 versus 39% of inoculated roots showing tumors). Analysis by the Student’s *t*-test confirmed that tumorigenesis of LBA1010 and LBA2564 on Arabidopsis Col-0 was significantly different. To determine whether overexpression of *virE3* in Arabidopsis could increase susceptibility to *Agrobacterium* infection, we used a transgenic line of Arabidopsis that expresses the *virE3* coding sequence under the control of the tamoxifen-inducible promoter (Niu et al., 2015). After induction by tamoxifen (Figure 2C) the roots of *virE3*-OE line1 were transformed by LBA1010 and LBA2564 and the fractions of roots that formed tumors were calculated. As shown in Figure 2B, the *virE3-*OE line1 was much more susceptible to *Agrobacterium* LBA1010 and LBA2564 infection with tumors forming in 52 and 41% of the inoculated roots for wild type and *virE3* mutant, respectively. Apparently, increasing the amount of VirE3 can enhance tumor formation even by the wild type *Agrobacterium* strain. The difference in tumorigenesis between LBA1010 and LBA2564 on *virE3-*OE line1 was statistically significant, suggesting that translocated VirE3 from LBA1010 also still contributes to tumor formation.

As we found that VirE3 can interact with JAZ8, we further investigated the role of JAZ8 in tumor formation by *Agrobacterium*. To this end, transgenic plant lines that constitutively expressed the *JAZ8* coding sequence under the CaMV 35S promoter were generated. Two independent T1 transgenic lines, designated *JAZ8-*OE1 and *JAZ8-*OE2, both showed about ninefold higher levels of *JAZ8* transcript accumulation in their roots compared with Col-0 wild type by qRT-PCR analysis. The roots of *JAZ8-*OE line1 were transformed by LBA1010 and LBA2564 and the fraction of roots on which tumors were formed was calculated. Interestingly, we found that on *JAZ8-*OE line1 much less tumors were formed by our wild type strain LBA1010 than on Arabidopsis Col-0 (27 versus 39% of inoculated roots showing tumors, statistically significant). When the *virE3* mutant LBA2564 was used for inoculation of the *JAZ8-*OE roots, the fraction of roots forming tumors was even lower with tumorigenesis seen on only about 22% of the roots (Figure 2B). Our results indicate that JAZ8 counters *Agrobacterium* virulence, whereas VirE3 enhances *Agrobacterium* tumorigenicity on Arabidopsis roots.

### JAZ8 Reduces Activity of VirE3 as a Transcriptional Activator

**FIGURE 3:**
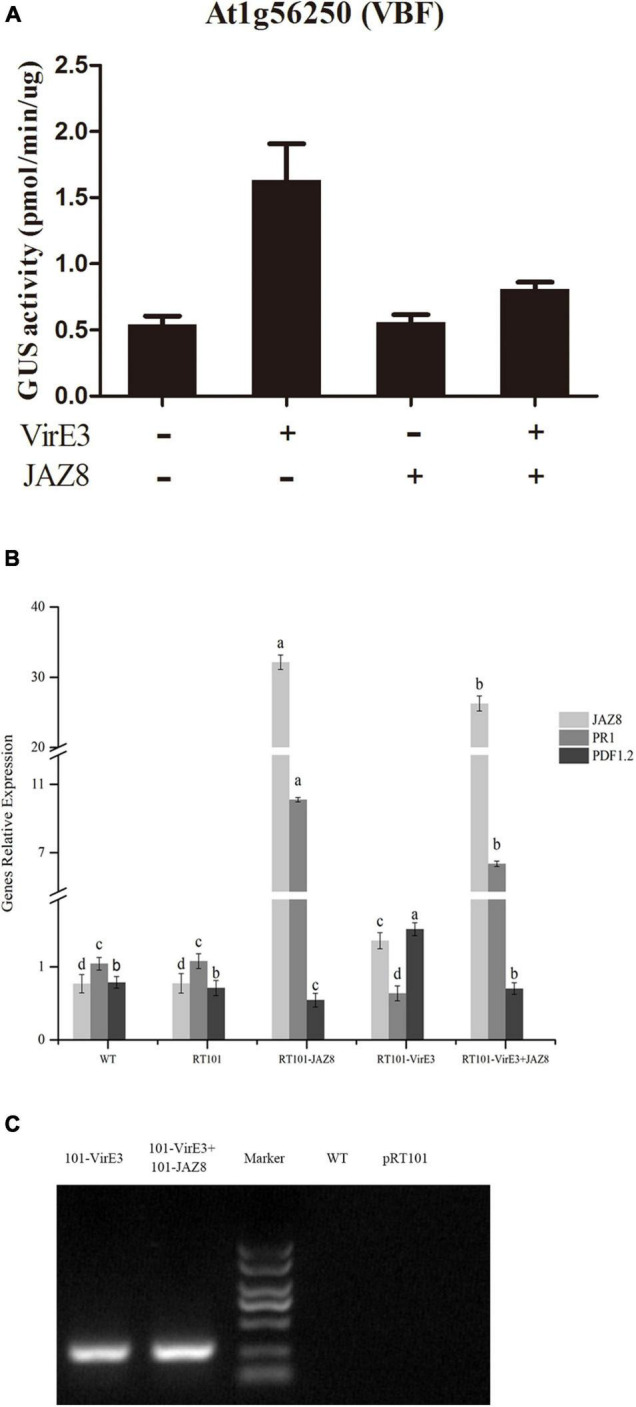
Protoplast expression analysis of the promoters of At1G56250 (*VBF*) and the gene expression analysis of *PR1*, *PDF1.2*, *JAZ8*, and *virE3*. **(A)** JAZ8 inhibits VirE3-dependent activation of the At1G56250 (*VBF*) (857 bp) promoters in Arabidopsis protoplasts. **(B)** qPCR analysis of the expression level of *PR1*, *PDF1.2*, *JAZ8* in protoplasts transfected with corresponding plasmids. **(C)** RT-PCR analysis of the expression of *virE3* in protoplast transformed with corresponding plasmids.

VirE3 can act as a plant transcription factor which activates genes such as Arabidopsis *VBF*, which are needed for tumorigenesis (Niu et al., 2015). JAZ8 belongs to the plant-specific TIFY family of transcriptional regulators that repress JA-regulated defense responses by binding to MYC transcription factors (Shyu et al., 2012). To find out whether JAZ8 could reduce the transcriptional activity of VirE3, the effect of JAZ8 on the VirE3-dependent activation of *VBF* (At1G56250) promoter was investigated in a protoplast transactivation assay. The 857 bp of *VBF* promoter was fused to the GUS reporter, and the full length of *virE3* or *JAZ8* coding sequences wase cloned into the pRT101 plasmid, in which *virE3* or *JAZ8* was expressed from the CaMV 35S promoter. Arabidopsis protoplasts were cotransformed with the *VBF*-promoter-GUS reporter plasmid and at the same time with the effector plasmid pRT101-VirE3 and/or pRT101-JAZ8. First we measured whether the *JAZ8* and *virE3* construct were expressed in Arabidopsis protoplasts by RT-PCR. As shown in Figure 3B, the mRNA expression level of *JAZ8* was around 30-fold higher in protoplasts transformed with plasmid pRT101-JAZ8 and also in protoplasts transformed by pRT101-JAZ8 together with pRT101-VirE3 compared with that in Col-0 protoplasts. The mRNA expression of *virE3* was also confirmed by RT-PCR analysis in protoplasts transformed with plasmid pRT101-VirE3 and plasmids pRT101-VirE3 together with pRT101-JAZ8 (Figure 3C). These results indicate that the mRNAs of both genes are indeed expressed in the transformed protoplasts and that the expression of the *JAZ8* gene does not negatively affect the expression of *virE3* and vice versa. Subsequently, we tested the effect of JAZ8 and VirE3 on the activity of the *VBF* promoter. As shown in Figure 3A, expression of JAZ8 alone barely affected the activity of the *VBF* promoter, whereas VirE3, as expected, activated the *VBF* promoter by approximately threefold. In contrast, co-expression of VirE3 and JAZ8 together led to a twofold reduced activity of the *VBF* promoter as compared with that in the presence of VirE3 alone. As JAZ8 is known to affect the JA response and promote the SA defense response, we also tested whether VirE3 could affect these defense responses. To this end, we measured the mRNA levels of *PR1* (SA response) and *PDF1.2* (JA response) in the same transformed protoplasts. Interestingly, we found that the expression level of *PR1* was reduced in protoplasts transformed with pRT101-VirE3 and elevated in protoplasts transformed with pRT101-JAZ8. Reversely, the expression level of *PDF1.2* was elevated in protoplasts transformed with pRT101-VirE3 and decreased in protoplasts transformed with pRT101-JAZ8 (Figure 3B). In summary, our results indicate that JAZ8 can repress the transcriptional activity of VirE3 on activating the expression of *VBF.* Also overexpression of VirE3 reduced *PR1* gene expression and elevated *PDF1.2* expression, whereas overexpression of *JAZ8* elevated *PR1* gene expression and reduced *PR1* expression.

### Effect of *JAZ8* Overexpression on the Arabidopsis Transcriptome

**FIGURE 4:**
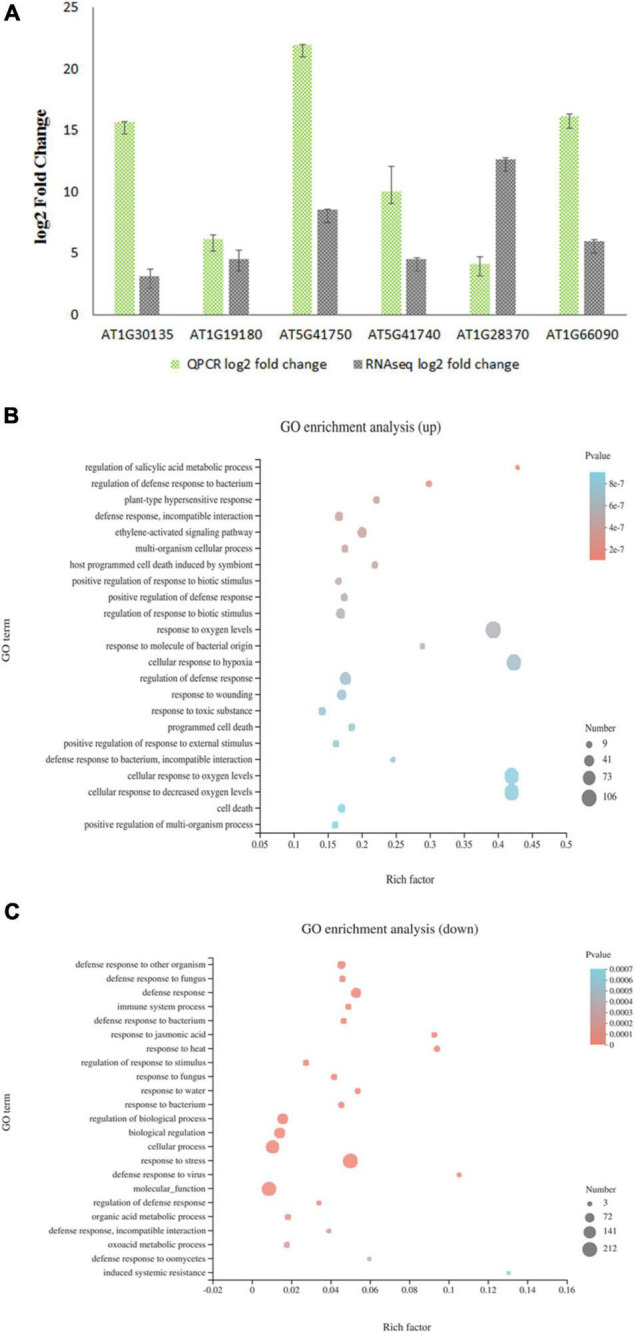
Transcript profiling of *AtJAZ8* overexpressor plants. **(A)** Validation of the RNA-Seq data by quantitative RT-PCR. Six differential expression genes in *AtJAZ8* overexpressor plants compared with control plant lines in the RNA-seq experiment were selected for validation by qRT-PCR. **(B)** GO term enrichment for 428 upregulated genes using Database for Annotation and Visualization. **(C)** GO term enrichment for 268 downregulated genes using Database for Annotation and Visualization.

As a transcriptional repressor of the jasmonate downstream signaling pathway, JAZ8 most likely affects plant susceptibility to *Agrobacterium* by regulating the expression of plant defense responsive genes that interfere with the *Agrobacterium* infection. To investigate the role of JAZ8 on the transcription of defense responsive genes, we carried out transcriptome profiling by comparing *AtJAZ8* overexpressor lines with control lines using RNA-seq analysis. Total RNA samples extracted from seedlings were analyzed for gene expression using RNA-seq. Using DESeq to calculate differentially expressed genes (DEGs), we identified a total of 696 genes that were either up- or down-regulated (FDR < 0.001 and log2FC > 2) in JAZ8-OE plant lines compared with the control lines (Supplementary Table 1). Raw data have been deposited in the NCBI SRA database (BioProject accession number: PRJNA719307). To validate the RNA-seq results, six DEGs from *AtJAZ8* overexpressor plants were randomly selected for analysis by quantitative RT-PCR. As shown in Figure 4A, the results obtained from qRT-PCR analysis are in general agreement with the RNA-seq results. We then used GO annotation (TAIR10) to assign genes to functional categories and performed function enrichment analysis on the DEGs. The 428 genes upregulated in the *JAZ8-*OE line were enriched for genes implicated in regulation of SA metabolic process, regulation of defense response to bacterium, regulation of plant-type hypersensitive response, and also defense response (Figure 4B). By contrast, the 268 genes downregulated in the *JAZ8*-OE line were enriched for genes related to defense response, immune system process, response to JA, etc. (Figure 4C). Our results suggest that JAZ8 generally affects genes involved in plant defense responses. Interestingly, we found that the expression level of pathogenesis-related gene 1 (*AtPR1*) in *JAZ8-*OE was around five times (5.48) higher than in control lines, indicating JAZ8 may activate the SA-mediated signaling pathway against *Agrobacterium* infection.

### JAZ8 Antagonizes VirE3 on Expression of *AtPR1*

**FIGURE 5:**
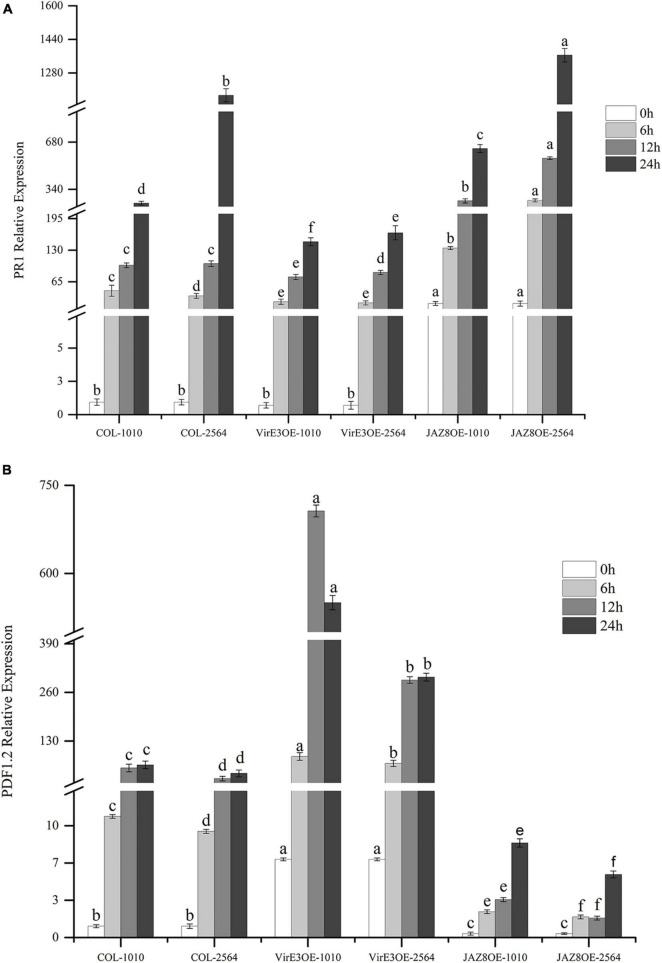
Quantification of *AtPR1* and *AtPDF1.2* gene expression level in Col-0, *virE3*-OE and *JAZ8*-OE1 lines after infection with *Agrobacterium* LBA1010 and LBA2564 at different time points. **(A)** qRT-PCR analysis of *AtPR1* gene expression pattern. **(B)** qRT-PCR analysis of *AtPDF1.2* gene expression pattern. *EF1a* was used as internal control for data normalization. The expression level of *PR1* and *PDF1.2* in the wild-type Col-0 at 0 h was set to 1.0, and error bars represent SEM of three independent biological replicates. The expression values of *PR1* and *PDF1.2* in *virE3*-OE and *JAZ8*-OE lines were compared to the control Col-0 at each corresponding condition. Differences indicated by different letters are statistically significant (*P* values < 0.05) and by the same letter are not statistically significant.

We have shown that the percentage of root tumors was highly decreased in *JAZ8-*OE lines and increased in *virE3*-OE lines. To investigate the plant defense genes responsible for *Agrobacterium* mediated root tumorigenesis, the expression level of Arabidopsis *pathogenesis-related GENE 1* (*AtPR1*) and Arabidopsis *PLANT DEFENSIN 1.2*(*AtPDF1.2*) were measured by qRT-PCR in wild type Col-0, *JAZ8-*OE, and *virE3-*OE plant lines infected by *Agrobacterium* strains LBA1100 (wild-type) and LBA2564 (*virE3* deletion mutant) at different time points. To compare the gene expression level at different time points infected by two *Agrobacterium* strains LBA1100 and LBA2564, we normalized the expression level of *AtPR1* and *AtPDF1.2* to the Col-0 at 0 h before infection by qRT-PCR analysis. As shown in Figure 5A, 24 h after *Agrobacterium* infection, the expression level of *AtPR1* was significantly reduced in *virE3*-OE plant line compared with that in Col-0 and *JAZ8*-OE plant line, whereas the expression level of *AtPR1* gene was the highest in the *JAZ8*-OE plant line. Our results showed that VirE3 inhibits the expression of *AtPR1* gene and JAZ8 promotes *AtPR1* gene expression probably by repressing the JA-responsive defense pathway. The expression pattern of *AtPR1* in plant lines was negatively well correlated with the root tumorigenicity by *Agrobacterium*. On the contrary, after *Agrobacterium* infection, the expression level of *AtPDF1.2* was highest in *virE3-*OE plant line and lowest in *JAZ8-*OE plant line, indicating VirE3 might activate the JA-induced signaling pathway by repressing *AtPR1* gene expression (Figure 5B). Our results showed that JAZ8 may antagonize VirE3 on activating the expression of *AtPR1* gene, which in turn attenuates *Agrobacterium* tumorigenesis on Arabidopsis root.

## Discussion

*Agrobacterium* delivers several virulence proteins, including VirD2, VirE2, VirE3, VirF, and VirD5 to plant cell for successful T-DNA integration and tumorigenesis (Lacroix and Citovsky, 2019). One of the translocated effector proteins, VirE3, is highly conserved among different *Agrobacterium* and rhizobia species, suggesting that VirE3 may play an important role during AMT. However, it was reported that VirE3 was not essential for AMT by octopine strains and a *virE3* mutation did not significantly affect tumor formation on the stems of several plant species, and its contribution to tumorigenesis could only be seen in *virE3 virF* double mutants (García-Rodríguez et al., 2006). A recent report showed that VirE3 is required for full virulence of the agropine/succinamopine strain EHA105, which does not have a close homolog of octopine Ti *virF*. Deletion of *virE3* resulted in a decreased transformation efficiency in the transient transformation assays of *Nicotiana benthamiana* leaves (Li et al., 2018, 2020). In contrast, another report showed constitutive overexpression of *virE2* or *virE3* under CaMV 35S promoter in Arabidopsis-induced plant defense responses and conferred resistance to *Agrobacterium* infection (Duan et al., 2018). Our results showed that VirE3 is required for tumorigenesis of oncogenic wild type *Agrobacterium* LBA1010. Deletion of *virE3* attenuated *Agrobacterium* virulence in root tumor assay, whereas transient overexpression of *virE3* enhanced *Agrobacterium* tumorigenesis (Figure 2A). The different results reported may be due to the different methods used for the transformation or to the different plant species or *Agrobacterium* strains used.

Upon attack by pathogens, plants possess highly sophisticated mechanisms to induce the expression of defense genes to restrict pathogen invasion. The phytohormones SA and JA, as key signaling molecules, play important roles in regulating the activation of the induced defense responses. Silencing of SA biosynthetic and signaling genes in *N. benthamiana* increases susceptibility to crown gall disease (Yuan et al., 2007; Anand et al., 2008). One of the crucial effects of SA in plant defense is to activate the expression of pathogenesis related genes such as *PR1*, resulting in both local and systemic acquired resistance to the pathogens. However, *Agrobacterium* has evolved strategies to dampen this host defense response. It has been reported that infection by *Agrobacterium* decreases the level of free SA in roots, leading to reduced *PR1* gene expression and a diminished systemic-acquired resistance (SAR) (Gaspar et al., 2004). Otherwise it has been reported that *Agrobacterium* infection triggers the activation of mitogen-activated protein kinase (MAPK) MPK3, which phosphorylates the transcription factor VIP1, leading to its relocalization from the cytoplasm to the nucleus, and also in the of induction of stress-related genes. This can indirectly lead to increased *PR1* gene expression (Djamei et al., 2007). However, the translocated effector VirE3 was shown to induce the expression of *NIMIN1* (Niu et al., 2015). The NIMIN1 protein binds to NPR1/NIM1 and prevents the induction of *PR1* and SAR (Niu et al., 2015). It has been shown that exogenous application of SA also affects *Agrobacterium* itself and inhibits induction of its virulence genes, and thus decreases its infectivity (Yuan et al., 2007). A recent publication revealed that SA is used by *Agrobacterium* to turn down the expression of its virulence genes late in infection. To this end *Agrobacterium* expresses the hydrolase SghA to liberate plant SA from the storage form, SA-glucoside (Wang et al., 2019).

Our new results showed that *VirE3* enhances *Agrobacterium* tumorigenesis by reducing *PR1* defense gene expression at the early stage (Figure 5A). Using yeast two-hybrid screening, we found that VirE3 can interact with Jasmonate ZIM domain (JAZ) repressor JAZ8 *via* its ZIM domain. JAZ proteins are suppressors of JA-induced transcriptional response, reducing the transcription of JA-responsive genes such as *PDF1.2*. Our results are in line with this and show that JAZ8 suppresses the JA-dependent defense response as seen by the downregulation of *PDF1.2*, and promotes the SA-dependent response as seen by enhanced *PR1* gene expression with increased levels of JAZ8 expression (Figure 5B). JAZ8-OE in turn attenuates Agrobacterium tumorigenesis as shown in Figure 2B. We found that the effector proteinVirE3 binds to and thereby counteracts the activity of JAZ8 and thus promotes tumorigenesis by *Agrobacterium*. In the presence of VirE3, the expression of JA-defense genes is increased and the expression level of SA-defense genes decreased.

In conclusion, our data shown herein revealed that the effector protein VirE3 is required for the full virulence of *Agrobacterium* by counteracting the transcriptional repressor activity of JAZ8, thus promoting the JA defense response and diminishing the SA defense response. Such a strategy is not uncommon for plant pathogenic bacteria. Plant pathogenic *Pseudomonas syringae* bacteria secrete coronatine, a jasmonate mimic, to enhance the JA defense response. Other strains may deliver Hop effector proteins *via* a Type3 secretion system such as HopZ1 that bind to and modify JAZ proteins leading to their destruction (Jiang et al., 2013). Whether *Agrobacterium* VirE3 binding to JAZ8 similarly leads to its degradation requires further study.

## Data Availability Statement

The original contributions presented in the study are publicly available. This data can be found here: NCBI repository, accession number: PRJNA719307 (https://www.ncbi.nlm.nih.gov/bioproject/PRJNA719307).

## Author Contributions

BX, MZ, XN, and PH conceived and designed the experiment. SL performed the experiments. XN and SL wrote the manuscript. MZ, XL, and JC provided critical analysis of the design and data. All authors read and approved the final manuscript.

## Conflict of Interest

The authors declare that the research was conducted in the absence of any commercial or financial relationships that could be construed as a potential conflict of interest.

## Publisher’s Note

All claims expressed in this article are solely those of the authors and do not necessarily represent those of their affiliated organizations, or those of the publisher, the editors and the reviewers. Any product that may be evaluated in this article, or claim that may be made by its manufacturer, is not guaranteed or endorsed by the publisher.

## Footnotes

1
http://login.majorbio.com/login
