# Supplementary material for: JAZ8 Interacts With VirE3 Attenuating Agrobacterium Mediated Root Tumorigenesis
Source: Front Plant Sci. 2021 Nov 19;12:685533. doi: 10.3389/fpls.2021.685533 (PMC8639510; doi:10.3389/fpls.2021.685533)
Supplement: Supplementary file 1 [file Data_Sheet_1.docx]

**Table S1. Primer sequences used in this study**

| **Primer Name** | **Sequence (5’ to 3’)** | **Purpose** |
| --- | --- | --- |
| JAZ8-1302-F | CCATGATTACGAATTCATGAAGCTACAGCAAAATTG | Cloning of *JAZ8-OE* construct |
| JAZ8-1302-R | GGCCAGTGCCAAGCTTTTATCGTCGTGAATGGTACG | Cloning of *JAZ8-OE* construct |
| JAZ8-pAS-F | GAATTCATGAAGCTACAGCAAAATTGTG | Cloning of *JAZ8* Y2H construct |
| JAZ8-pAS-R | GTCGAC TTATCGTCGTGAATGGTACG | Cloning of *JAZ8* Y2H construct |
| virE3-pACT-F | CCATGGATGGTGAGCACTACGAAGAAAAG | Cloning of *virE3* Y2H construct |
| virE3-pACT-R | GGATCCCCGAACAACGATTGCGGGTC | Cloning of *virE3* Y2H construct |
| JAZ8NT-pAS-F | CCATGGGGAACTTCGTCTT | Cloning of *JAZ8NT* Y2H construct |
| JAZ8NT-pAS-R | GAATTCTAGCCCGTTTGAGGATGACTTG | Cloning of *JAZ8NT* Y2H construct |
| JAZ8CT-pAS-F | CCATGGTCCAAAAGCATCAATGAAAAAATC | Cloning of *JAZ8CT* Y2H construct |
| JAZ8CT-pAS-R | GGATCCGTACGGTGAAGTAGCTTGAATTCG | Cloning of *JAZ8CT* Y2H construct |
| JAZ8ZIM-pAS-F | CCATGGAATAACAATTTTCTACAATGG | Cloning of *JAZ8ZIM* Y2H construct |
| JAZ8ZIM-pAS-R | GAATTCTAGGGAAGCTGATTATGATGAAATGAGG | Cloning of *JAZ8ZIM* Y2H construct |
| JAZ8-c5X-F | GAAGGATTTCACATATGATGAAGCTACAGCAAAATTGTG | Cloning of *JAZ8* Pull-down construct |
| JAZ8-c5X-R | CAGGGAATTCGGATCCTTATCGTCGTGAATGGTACGG | Cloning of *JAZ8* Pull-down construct |
| virE3-pGEX-6P-F | GGGGCCCCTGGGATCCATGGTCAGCATCCTGAAAAG | Cloning of *virE3* Pull-down construct |
| virE3-pGEX-6P-R | GGCCGCTCGAGTCGACCTAAAGCGTTACCGCCGATG | Cloning of *virE3* Pull-down construct |
| JAZ8-cYFP-F | ACCATGGAGATGAAGCTACAGCAAAATTGT G | Cloning of *JAZ8* BIFC assay construct |
| JAZ8-cYFP-R | GCAAGCGGCCGCGTTCGTCGTGAATGGTACGGTGAAG | Cloning of *JAZ8* BIFC assay construct |
| virE3-nYFP-F | ACCATGGAGATGGTGAGCACTACGAAG | Cloning of *virE3* BIFC assay construct |
| virE3-nYFP-R | GCAAGCGGCCGCGTGAAACCTCTGGAGGTGGAACG | Cloning of *virE3* BIFC assay construct |
| pRT101-JAZ8-F | AGGACCTCGAGAATTCATGAAGCTACAGCAAAATTGTGACTT | Cloning of *JAZ8* transient overexpression vector |
| pRT101-JAZ8-R | TTTTGCGGACTCTAGATTATCGTCGTGAATGGTACGGTG | Cloning of *JAZ8* transient overexpression vector |
| JAZ8-Q-F | TATCGATCGCAAGCAGAGAA | Real-time PCR |
| JAZ8-Q-R | TCGTGAATGGTACGGTGAAG | Real-time PCR |
| virE3-Q-F | CGGTGACGCAGGAGGTCTATT | Real-time PCR |
| virE3-Q-F | GCGGATCAGGTCGCCATTTT | Real-time PCR |
| EF1a-F (At5G09810) | TCCTTCTTGTCCACGCTCTT | Real-time PCR |
| EF1a-R (At5G09810) | TGGTGACGCTGGTATGGTTA | Real-time PCR |
| PR-1QF (AT2G14610) | GCTCTTGTTCTTCCCTCGAAAG | Real-time PCR |
| PR-1QR (AT2G14610) | AAGTCACCGCTACCCCAG | Real-time PCR |
| PDF1.2QF (AT5G44420) | TCACCCTTATCTTCGCTGCTCT | Real-time PCR |
| PDF1.2QR (AT5G44420) | AATACACACGATTTAGCACC | Real-time PCR |

^a^Restriction sites are underlined.

**Table S2. Plasmids and strains used in this study**

| **Name** | **Description** | **Antibiotic resistance^a^** | **Reference** |
| --- | --- | --- | --- |
| pCAMBIA1302 | Binary vector for plant transformation | Kan | CAMBIA |
| JAZ8-1302 | Binary vector containing a *JAZ8* coding sequence driven by a double CaMV 35S promoter. | Kan | This study |
| pAS2.1 | Yeast two hybrid vector with GAL4 DNA binding domain | Amp | ([James et al., 1996](#_ENREF_3)) |
| pACT2 | Yeast two hybrid vector with GAL4 DNA activation domain | Amp | ([James et al., 1996](#_ENREF_3)) |
| JAZ8-pAS2.1 | Plasmid containing the GAL4 DNA binding domain-tagged-JAZ8 fusion protein | Amp | This study |
| JAZ8NT-pAS2.1 | Plasmid containing the GAL4 DNA binding domain-tagged-JAZ8NT fusion protein | Amp | This study |
| JAZ8CT-pAS2.1 | Plasmid containing the GAL4 DNA binding domain-tagged-JAZ8CT fusion protein | Amp | This study |
| JAZ8ZIM-pAS2.1 | Plasmid containing the GAL4 DNA binding domain-tagged-JAZ8ZIM fusion protein | Amp | This study |
| virE3-pACT2 | Plasmid containing the GAL4 DNA activation domain-tagged-virE3 fusion protein | Amp | This study |
| pGEX-6P-1 | Bacterial expression vector with an N-terminal GST tag | Amp | This study |
| virE3-GST | plasmid containing the GST-tagged-JAZ8 fusion protein | Amp | This study |
| pMAL-c5X | Bacterial expression vector with a C-terminal MBP tag | Amp | This study |
| JAZ8-MBP | plasmid containing the MBP-tagged-JAZ8 fusion protein | Amp | This study |
| pRTL2-HAYC | BiFc vector with a C-terminal (YC) fragment of yellow fluorescent protein (YFP) | Amp | ([Bracha-Drori et al., 2004](#_ENREF_1)) |
| JAZ8-cYFP | plasmid containing the C-terminal part of the yellow fluorescent-JAZ8 fusion protein | Amp | This study |
| pRTL2-EEYN | BiFc vector with a N-terminal (YN) fragment of yellow fluorescent protein (YFP) | Amp | ([Bracha-Drori et al., 2004](#_ENREF_1)) |
| virE3-nYFP | plasmid containing the N-terminal part of the YFP-virE3 fusion protein | Amp | This study |
| pRT101 | Plant transient expression vector driven by a double CaMV 35S promoter | Amp | ([Töpfer et al., 1987](#_ENREF_5)) |
| pRT101-JAZ8 | plasmid containing the *JAZ8* coding sequence driven by a double CaMV 35S promoter | Amp | This study |
| pRT101-virE3 | plasmid containing the *virE3* coding sequence driven by a double CaMV 35S promoter | Amp | ([Niu et al., 2015](#_ENREF_4)) |
| VBF-promoter-GUS | plasmid containing the *GUS* coding sequence driven by VBF promoter | Amp | ([Niu et al., 2015](#_ENREF_4)) |

**Table S3. Bacterial strains used in this study**

| **Strain name** | **Description** | **Reference** |
| --- | --- | --- |
|  | ***E. coli* strains** |  |
| DH5α | Host for DNA cloning | This study |
| BL21 (DE3) pLysS | Host for protein expression | This study |
|  | ***Agrobacterium* strains** |  |
| LBA1010 | Tumorigenic | ([García-Rodríguez et al., 2006](#_ENREF_2)) |
| LBA2564 | Tumorigenic | ([García-Rodríguez et al., 2006](#_ENREF_2)) |
| GV3101 | JAZ8 in GV3101 | This study |
|  | **Yeast strains** |  |
| PJ694A | Yeast 2 Hybrid | ([James et al., 1996](#_ENREF_3)) |

**Reference**

Bracha-Drori, K., Shichrur, K., Katz, A., Oliva, M., Angelovici, R., Yalovsky, S., et al. (2004). Detection of protein-protein interactions in plants using bimolecular fluorescence complementation. *Plant J* 40(3)**,** 419-427. doi: 10.1111/j.1365-313X.2004.02206.x.

García-Rodríguez, F.M., Schrammeijer, B., and Hooykaas, P.J.J. (2006). The Agrobacterium VirE3 effector protein: a potential plant transcriptional activator. *Nucleic Acids Research* 34(22)**,** 6496-6504. doi: 10.1093/nar/gkl877.

James, P., Halladay, J., and Craig, E.A. (1996). Genomic libraries and a host strain designed for highly efficient two-hybrid selection in yeast. *Genetics* 144(4)**,** 1425-1436.

Niu, X., Zhou, M., Henkel, C.V., van Heusden, G.P.H., and Hooykaas, P.J.J. (2015). TheAgrobacterium tumefaciensvirulence protein VirE3 is a transcriptional activator of the F-box geneVBF. *The Plant Journal* 84(5)**,** 914-924. doi: 10.1111/tpj.13048.

Töpfer, R., Matzeit, V., Gronenborn, B., Schell, J., and Steinbiss, H.-H. (1987). A set of plant expression vectors for transcriptional and translational fusions. *Nucleic Acids Research* 15(14)**,** 5890-5890. doi: 10.1093/nar/15.14.5890.
